# Accelerated 3D MRI for ARIA monitoring in Alzheimer's disease

**DOI:** 10.1002/alz.71297

**Published:** 2026-03-24

**Authors:** Miguel Rosa‐Grilo, Dermot Mallon, David L. Thomas, Millie Beament, Haroon R. Chughtai, Wei Liu, Nicholas Magill, Ian B. Malone, Heiko Meyer, Geoff J. M. Parker, Christina Triantafyllou, Frederik Barkhof, Nick C. Fox, Catherine J. Mummery

**Affiliations:** ^1^ Dementia Research Centre, UCL Queen Square Institute of Neurology University College London London UK; ^2^ Lysholm Department of Neuroradiology National Hospital for Neurology and Neurosurgery London UK; ^3^ UCL Hawkes Institute University College London London UK; ^4^ Department of Translational Neuroscience and Stroke, UCL Queen Square Institute of Neurology University College London London UK; ^5^ Department of Medical Physics and Biomedical Engineering University College London London UK; ^6^ Advanced Research Computing (ARC) Centre University College London London UK; ^7^ Research & Clinical Translation Magnetic Resonance, Siemens Healthineers AG Erlangen Germany; ^8^ Department of Medical Statistics London School of Hygiene & Tropical Medicine London UK; ^9^ Bioxydyn Limited Manchester UK; ^10^ Research and Scientific Collaboration Siemens Healthcare Ltd Camberley UK; ^11^ Department of Radiology and Nuclear Medicine Amsterdam UMC & Vrije Universiteit Amsterdam The Netherlands

**Keywords:** advanced parallel imaging, Alzheimer's disease, amyloid‐related imaging abnormalities, MRI acceleration techniques, structural MRI, Wave‐CAIPI

## Abstract

**INTRODUCTION:**

Amyloid‐targeting therapies for Alzheimer's disease require regular MRI monitoring for amyloid‐related imaging abnormalities (ARIA). 3D scans are more sensitive but time intensive; ultra‐fast implementations could improve access and reduce burden.

**METHODS:**

Eighty scans from 20 participants were acquired with standard 2D fluid‐attenuated inversion recovery (FLAIR) and T2*‐gradient recalled echo (T2*‐GRE), or accelerated Wave‐controlled aliasing in parallel imaging (Wave‐CAIPI) 3D FLAIR and susceptibility‐weighted imaging (SWI) at 3 T. Two neuroradiologists graded ARIA‐E (edema/effusion) and ARIA‐H (hemosiderin deposits). Bayesian models estimated sensitivity, specificity, severity agreement, and interchangeability between acquisitions.

**RESULTS:**

Accelerated sequences reduced acquisition time by up to 56%. Four participants had ARIA‐E and microbleeds; five had microbleeds alone. Sensitivity and specificity for ARIA‐E were identical (1.00; 0.94–0.95); severity was comparable. Replacing standard with accelerated FLAIR did not decrease severity agreement (interchangeability 1.4; 95% highest‐density interval [HDI] −3.6% to 5.4%). Fast SWI showed higher microbleed severity gradings.

**DISCUSSION:**

Wave‐CAIPI offers fast high‐resolution FLAIR acquisitions with comparable performance for ARIA‐E monitoring. Wave‐CAIPI SWI provides high‐quality scans that may aid ARIA‐H interpretation.

## Highlights

- Wave‐controlled aliasing in parallel imaging (Wave‐CAIPI) sequences cut acquisition time by up to 56%
- Wave‐CAIPI FLAIR delivers fast, high‐resolution images with ARIA‐E) safety monitoring performance comparable to conventional 2D FLAIR
- Wave‐CAIPI SWI preserves enhanced sensitivity for microbleed detection while maintaining excellent image quality
- Fast protocols may improve access to MRI and Alzheimer's disease treatment programs

## 1 BACKGROUND

Alzheimer's disease (AD) treatment has entered a new era with the emergence of effective disease‐modifying therapies (DMTs).^1^, ^2^ In this evolving therapeutic paradigm, structural brain magnetic resonance imaging (MRI) is essential to assess treatment eligibility and monitor safety, and underpins the safe deployment of amyloid‐targeting DMTs in clinical settings.^3^, ^4^

Amyloid‐related imaging abnormalities (ARIA) are MRI‐detected adverse effects of amyloid‐targeting DMTs.^5^ Two subtypes are recognized: ARIA‐E (edema/effusion), visualized as transient hyperintensities on fluid‐attenuated inversion recovery (FLAIR) sequences without restricted diffusion; and ARIA‐H (hemosiderin deposits), comprising parenchymal microbleeds and superficial siderosis, detected on heme‐sensitive sequences such as T2*‐weighted gradient recalled echo (T2*‐GRE) or susceptibility‐weighted imaging (SWI).^5^, ^6^ Radiographic severity scales classify ARIA‐E and each ARIA‐H component as mild, moderate or severe, and are central to clinical management alongside symptom severity.^3^, ^4^, ^7^, ^8^, ^9^ Since the vast majority of ARIA are asymptomatic and first detected on surveillance MRI,^1^, ^2^ and are more likely to occur early in the course of treatment, current guidelines recommend a baseline MRI followed by three to four scans in the first 6 months of treatment.^3^, ^4^ Additional MRI scans are advised for new or worsening symptoms concerning ARIA and for ARIA follow‐up,^3^, ^4^ with longer‐term surveillance schedules less well established.^8^ Core sequences in surveillance protocols include 2D / 3D FLAIR and axial T2*‐GRE, preferably at 3 T, which has been favored due to its wide availability.^10^ The increased sensitivity of SWI to heme products^11^ may have a role in aiding interpretation.^10^

Limited access to magnetic resonance imaging (MRI) is a bottleneck to the widespread implementation of these new therapies.^12^, ^13^ Even in advanced economies, MRI capacity remains limited.^14^ Surveys of clinical practice show that for diagnosis, many patients do not receive an MRI despite being the imaging modality of choice.^15^, ^16^ Audits of clinical services in England have found wide variation between sites;^17^ of brain scans performed, only 31.8% were MRI.^18^ In the United States, a study of Medicare beneficiaries showed that fewer than 18% of individuals with cognitive symptoms underwent MRI,^19^ possibly reflecting MRI scanner capacity, and payer‐ or patient‐level access barriers.

The size of the AD population^20^—far exceeding that of most other neurological diseases necessitating brain imaging^21^, ^22^—places immense pressure on imaging services if MRI is to become the standard‐of‐care, underscoring the need for innovative and efficient scanning strategies. Advances in imaging acceleration offer a promising solution, typically requiring no hardware upgrade: faster acquisitions and streamlined protocols can improve patient comfort, reduce motion artifacts, and increase throughput, thereby lowering wait times and costs.^23^ Wave‐controlled aliasing in parallel imaging (Wave‐CAIPI) is one such technique, using a modified k‐space trajectory to achieve high acceleration in three‐dimensional (3D) acquisitions.^24^, ^25^, ^26^ Studies show that Wave‐CAIPI FLAIR and SWI achieve diagnostic performance comparable to conventional implementations in AD,^27^ multiple sclerosis,^28^ and vascular disease,^29^, ^30^ but its role in ARIA safety monitoring remains unexplored.

Here, we evaluated the performance of an accelerated protocol using Wave‐CAIPI FLAIR and SWI compared to standard sequences for ARIA safety monitoring. Our aim was to apply Wave‐CAIPI acceleration to 3D acquisitions to achieve acquisition times shorter than standard 2D protocols, thereby enabling evaluation of a 3D approach that is feasible for routine ARIA monitoring.

## 2 METHODS

### 2.1 Study population

In this prospective study, we recruited 20 participants enrolled in AD clinical trials at the National Hospital for Neurology and Neurosurgery, United Kingdom, between October 2023 and September 2024. Specifically, we identified and approached individuals that were undergoing serial MRI surveillance for treatment as per trial protocols. Ethical approval was granted by the NHS Health Research Authority London (REC reference 21/LO/0815). The study was conducted in accordance with the Declaration of Helsinki of 1964 and its subsequent amendments. At enrollment, the median age of the participants was 45.5 (range 26–76). Participants had a diagnosis of familial or sporadic AD, and eight were female. At the trial baseline, the median mini‐mental examination state score was 27 (range 17–30), the median microbleed count was 0 (range 0–2), and the median Fazekas score for deep white matter hyperintensities was 0 (range 0–2).

**RESEARCH IN CONTEXT**

1. **Systematic review**: The authors reviewed the literature using databases such as PubMed and conference abstracts and presentations. Wave‐controlled aliasing in parallel imaging (Wave‐CAIPI) FLAIR and SWI have been evaluated in studies of white matter hyperintensities and cerebral microbleeds, but to our knowledge, no prior research has examined highly accelerated implementations for amyloid‐related imaging abnormalities (ARIA) safety monitoring in Alzheimer's disease.
2. **Interpretation**: Wave‐CAIPI FLAIR offers fast high‐resolution acquisitions with comparable performance to contemporaneous 2D FLAIR for ARIA‐E safety monitoring. Wave‐CAIPI SWI retains enhanced sensitivity to microbleed detection and has excellent image quality.
3. **Future directions**: Our findings support further evaluation of accelerated 3D magnetic resonance imaging (MRI) techniques, including Wave‐CAIPI, as time‐efficient acquisitions for ARIA safety monitoring. Additional research is needed to clarify the role of highly accelerated SWI.

### 2.2 Image acquisition

Scanning was performed on a 3 T MRI scanner (MAGNETOM Prisma, Siemens Healthineers, Forchheim, Germany) using a 64‐channel array receiver coil. Wave‐CAIPI sequences were provided by Siemens Healthineers as a research package to enable accelerated acquisitions, hereafter referred to as the fast protocol. All participants underwent imaging with the fast (3D FLAIR and SWI) and contemporaneous standard (2D FLAIR and T2*‐GRE) scan protocols during the same surveillance session, without distortion of the clinical trial safety monitoring schedule. Sequences from the two protocols were acquired in an interleaved manner (see Table 1 for sequence parameters). We have described the optimization of the fast sequences for clinical utility elsewhere.^27^

**TABLE 1:** Acquisition parameters for clinical and fast protocols

Sequence	Characteristic	Standard protocol^*^	Fast protocol
2D FLAIR / 3D FLAIR	Resolution	0.9 × 0.9 × 5.0 mm^3^	1.1 × 1.1 × 1.1 mm^3^
	Orientation	Transverse	Sagittal
	Number of slices	27	176
		35	
	Parallel imaging	GRAPPA x2	Wave‐CAIPI 3 × 2
	TI/TE/TR	2500 ms/96 ms/10000 ms	1800 ms/393 ms/5000 ms
		2500 ms/91 ms/9000 ms	
	Turbo factor	16	242
		18	
	Scan time	2 min 40s	1 min 59s
		4 min 05s	
T2*‐GRE/ SWI	Resolution	0.9 × 0.9 × 5.0 mm^3^	0.8 × 0.8 × 2.1 mm^3^
		0.8 × 0.8 × 4.0 mm^3^	
	Orientation	Transverse	Transverse
	Number of slices	27	72
		44	
	Parallel imaging	GRAPPA x2	Wave‐CAIPI 3 × 2
	TE/TR/flip angle	20 ms/639 ms/20°	21 ms/30 ms/15°
		20 ms/650 ms/20°	
	Scan time	2 min 20s	1 min 39s
		4 min 11s	

Abbreviations: 2D, two‐dimensional; 3D, three‐dimensional; FLAIR, fluid‐attenuated inversion recovery; GRAPPA, GeneRalized Autocalibrating Partially Parallel Acquisition; SWI, susceptibility weighted imaging; T2*‐GRE, T2*‐weighted gradient recalled echo; TE, echo time; TI, inversion time; TR, repetition time; Wave‐CAIPI, Wave‐controlled aliasing in parallel imaging.

* Multiple entries represent acquisition parameter variation across clinical trials. Standard protocol: 2D FLAIR and T2*‐GRE (5 to 8 min 16s); fast protocol: 3D FLAIR and SWI (3 min 38s).

### 2.3 Image evaluation

The gold‐standard assessment for ARIA was defined as the evaluation of the complete series of surveillance MRI scans, performed using the standard 2D FLAIR and T2*‐GRE acquisitions—the current industry standard for clinical trials. Specifically, for each of the two surveillance sessions selected per individual, ARIA‐E and ARIA‐H were assessed against the baseline scan (standard 2D FLAIR and T2*‐GRE protocol); any surveillance sessions available between each selected surveillance session and the baseline scan were used for adjudication where appropriate. The two surveillance sessions were selected a priori to include a mix of ARIA‐positive and ARIA‐negative examinations, where available, and selection was performed prior to ARIA ratings.

Separately, two consultant neuroradiologists with expertise in ARIA independently reviewed each surveillance scan protocol (standard or fast) against the baseline standard scan only (the fast protocol was not available at baseline). The 80 de‐identified scans (20 patients  × 2 protocols × 2 sessions per neuroradiologist) were presented in random order, with at least four cases from other participants separating scans of the same individual. Raters were not provided with information about the design of the image assessment process, demographics, number of sessions per individual, or protocol type. Standard radiographic severity scales^7^, ^8^ were used to grade ARIA‐E and ARIA‐H (microbleeds and superficial siderosis). In addition, we asked neuroradiologists to count new microbleeds, and rate image quality of each sequence using a predefined five‐point Likert scale (1 = non‐diagnostic featuring strong artifacts, 5 = excellent quality without artifacts; see Table S1 for rating scales). When neuroradiologists disagreed on ARIA severity for a given session/protocol (fast or standard) combination, a consensus rating was subsequently obtained by joint image review and discussion.

### 2.4 Statistical analysis

To estimate sensitivity and specificity of the neuroradiologists’ assessments at the detection level (i.e., presence or absence of ARIA), we modeled the four possible outcomes in a confusion matrix—true positives, false positives, true negatives, and false negatives relative to the gold‐standard assessments—using a multinomial logistic regression framework.^31^ Sensitivity and specificity were then calculated from the estimated category probabilities. For ARIA severity ratings, we defined the outcome as the discrepancy between their ratings and the gold‐standard assessments (i.e., assignment of lower ARIA severity, same severity, higher severity relative to gold‐standard). The modeling was performed using a cumulative ordinal model with a flexible threshold parameter. We fitted separate models for each ARIA type and analysis (detection, severity), including protocol type (standard, fast), rater, and their interaction as fixed effects, and a random intercept for patients to address the non‐independence of outcome measures. We repeated these models including image quality as an additional covariate to evaluate its influence on detection and severity ratings. We also refitted the models using consensus ratings in place of the individual assessments.

For the evaluation of interchangeability^32^ between standard and fast FLAIR for ARIA‐E, we assessed the pairwise agreement at the participant‐session level between neuroradiologists and protocols. Specifically, we estimated (1) the probability of agreement between the neuroradiologists’ assessments using standard acquisitions, (2) the probability of agreement between protocols, and (3) the individual equivalence index, defined as the relative frequency with which participants received a similar ARIA diagnosis on fast versus standard scans compared with the frequency of agreement when the same standard scan was assessed on two occasions. Two models were fitted: one for ARIA‐E detection (Bernoulli likelihood with a logit link) and one for ARIA‐E severity (cumulative ordinal model with a flexible threshold parameter). For severity, weighted agreement scores were used to account for partial matches, assigning a weight of 1 for exact agreement, 0.5 for adjacent severity categories, and 0 for larger discrepancies. The models included the pairwise protocol combination, the pairwise rater combination, and their interaction as covariates, and a random intercept for patients. Similarly to the previous analyses, we repeated these models including the pairwise absolute difference of image quality scores as an additional covariate. Finally, to estimate the incidence rate ratio of new microbleed occurrences on the fast scan relative to the standard acquisition, we fitted a Poisson model with protocol type, rater, and interval time since the baseline scan as covariates, and a random intercept for patients.

Models were fitted using the R Statistical language (version 4.5.1, R Core Team, 2025) on macOS using the brms^33^ package (version 2.22.13) with the cmdstanr interface (version 0.9.0.9000) to Stan for Bayesian estimation. For detection and severity relative to the gold‐standard assessments, we report (1) the posterior median probability for each protocol (standard, fast), (2) the 95% highest‐density interval (HDI) for the difference between protocols (standard—fast), and (3) the proportion of the entire posterior distribution of that difference lying within the region of practical equivalence (ROPE) of ± 0.05 absolute probability, computed with bayestestR (0.16.1).^34^ For the interchangeability analysis, we present the posterior median probabilities of agreement within standard acquisitions and between protocols, along with the 95% HDI for their difference (within standard—between protocols; individual equivalence index) and its proportion within the ROPE. For the Poisson model, we report the posterior median incidence rate ratio (IRR) and its 95% HDI (see Table S2 for further details).^35^, ^36^, ^37^, ^38^

## 3 RESULTS

**FIGURE 1:**
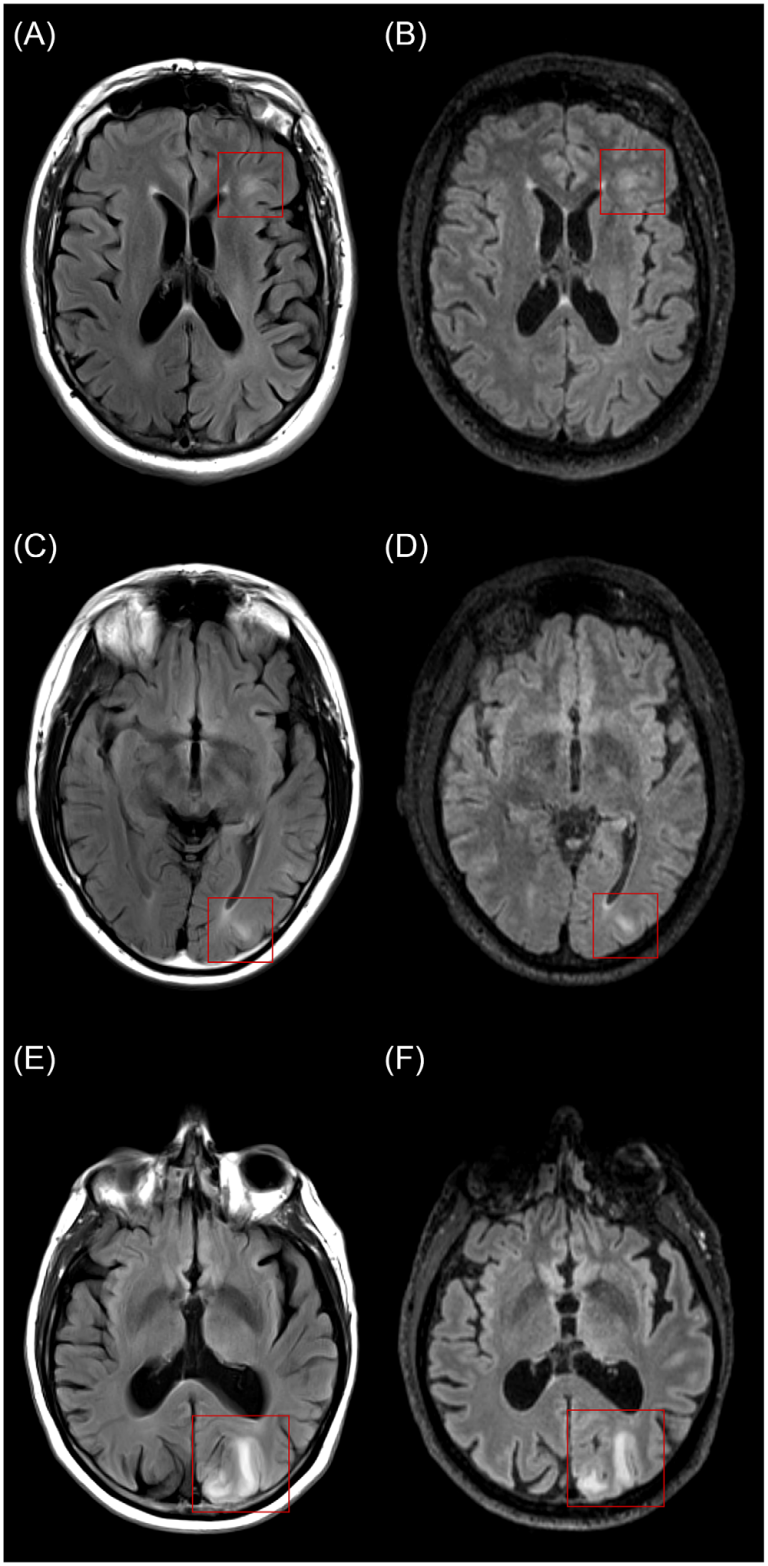
ARIA‐E. Panels (A), (C), and (E) show standard 2D FLAIR acquisitions (spatial resolution = 0.9 × 0.9 × 5mm^3^), and panels (B), (D), and (F) show fast 3D FLAIR acquisitions (spatial resolution = 1.1 × 1.1 × 1.1mm^3^). ARIA‐E regions are indicated by red squares. 2D, two‐dimensional; 3D, three‐dimensional; ARIA‐E, amyloid‐related imaging abnormalities with edema/effusion; FLAIR, fluid‐attenuated inversion recovery.

**FIGURE 2:**
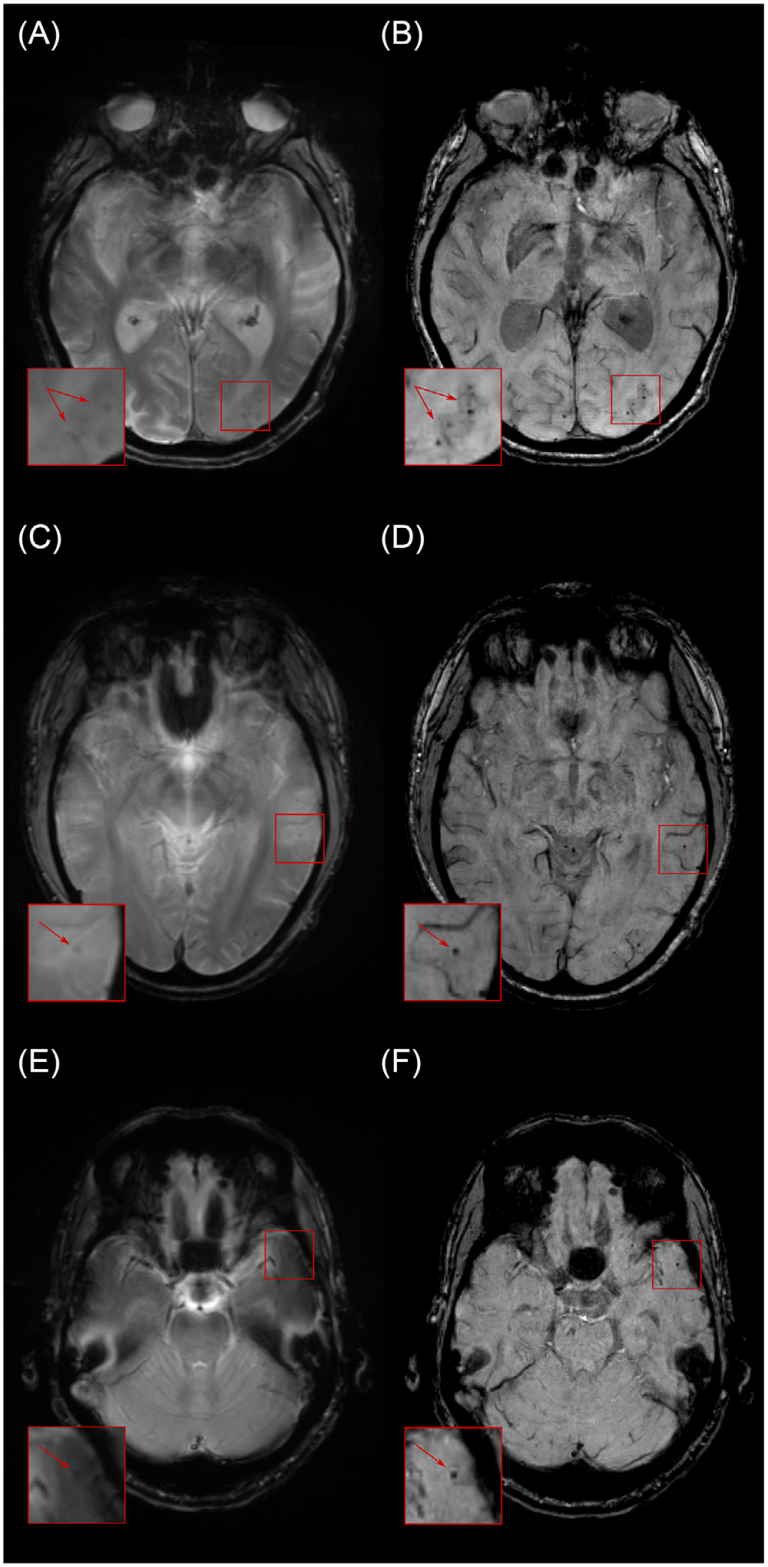
ARIA‐H. Panels (A), (C), and (E) show standard T2*‐GRE acquisitions; panels (B), (D), and (F) show fast SWI acquisitions. Magnified (×2) insets highlight regions containing microbleeds (arrows). Microbleeds are often less conspicuous on standard scans, where they may be obscured by artifacts. ARIA‐H, amyloid‐related imaging abnormalities with hemorrhage; T2*‐GRE, T2*‐weighted gradient recalled echo; SWI, susceptibility‐weighted imaging.

Based on the gold‐standard assessments, four individuals had ARIA‐E and new microbleeds detected in at least one of their scans. Five others had new microbleeds detected in isolation (without ARIA‐E). There were no cases with (incident) superficial siderosis. ARIA‐E severity ranged from 1 (mild) to 2 (moderate), and ARIA‐H microbleed severity ranged from 1 (mild) to 3 (severe). The maximum observed microbleed count was 21. Relative to the standard 2D protocol, the fast 3D protocol reduced the acquisition time by 56% (by 4 min 38s) in 85% and 27% (by 1 min 22s) in 15% of cases (depending on the duration of the standard protocol). Figures 1 and 2 show examples of ARIA‐E and ARIA‐H from standard and fast acquisitions.

Relative to the gold‐standard, the neuroradiologists’ assessments showed no false‐negatives for ARIA‐E for standard and fast acquisitions (see Table 2, Q1). Specificity was very similar (94% vs 95%; −1.2 percentage point difference; 95% HDI −5.5 to 3.0; 95.5% of the difference between protocols within ± 0.05 percentage points). Microbleed detection was consistent between protocols (sensitivity 91% vs 91%; specificity 99% vs 96%), though the 95% HDI for their difference was wider than that observed for ARIA‐E.

**TABLE 2:** ARIA detection and severity for standard and fast protocols.

**Type of ARIA**	**Standard scans** ^*^	**Fast scans** ^*^	**% Difference (95% HDI)**	**% in ROPE**
**Q1. How accurately did standard and fast scans detect presence/absence of ARIA relative to serial‐imaging gold‐standard assessments?**				
ARIA‐E				
Sensitivity	1.00	1.00	0.0 (0.0, 0.0)	100.0
Specificity	0.94	0.95	−1.2 (−5.5, 3.0)	95.5
ARIA‐H (microbleeds)				
Sensitivity	0.91	0.91	−0.7 (−13.0, 11.5)	59.8
Specificity	0.99	0.96	2.8 (−1.5, 8.9)	77.8
**Q2. How consistent were ARIA severity ratings from standard and fast scans relative to serial‐imaging gold‐standard assessments?**				
ARIA‐E				
Lower severity	0.03	0.07	−3.5 (−8.0, 0.8)	75.2
Same severity	0.91	0.90	1.1 (−1.6, 4.7)	97.7
Higher severity	0.06	0.03	2.1 (−0.5, 5.4)	95.2
ARIA‐H (microbleeds)				
Lower severity	0.14	0.06	8.6 (2.2, 16.1)	13.7
Same severity	0.83	0.85	−1.6 (−8.2, 4.6)	83.9
Higher severity	0.02	0.09	−6.8 (−12.2, −2.1)	22.6

**Type of ARIA**	**Within standard acquisitions** ^*^	**Between protocols** ^*^	**% Difference** ^†^ **(95% HDI)**	**% in ROPE**
**Q3. Can standard and fast scans be used interchangeably for the detection of ARIA?**				
ARIA‐E				
Agreement	0.95	0.97	−1.9 (−7.9, 2.9)	83.9
**Q4. Can standard and fast scans be used interchangeably for grading ARIA severity?**				
ARIA‐E				
Agreement	0.97	0.95	1.4 (−3.6, 5.4)	96.0

Abbreviations: ARIA, amyloid‐related imaging abnormalities; ARIA‐E, amyloid‐related imaging abnormalities with edema/effusion; ARIA‐H, amyloid‐related imaging abnormalities with hemorrhage; HDI, high density intervals; ROPE, region of practical equivalence.

* Values represent posterior medians on the probability scale from Bayesian logistic mixed‐effects models.

^†^ Individual equivalence index.

When benchmarked against the gold‐standard, the neuroradiologists’ ARIA‐E severity gradings were similar for both protocols (probability of assigning the same severity 0.91 vs 0.90; 1.1 percentage point difference; 95% HDI −1.6 to 4.7; 97.7% of the difference between protocols within ± 0.05 percentage points; see Table 2, Q2). Fast scans, however, had a slight tendency to assign lower severity than standard scans relative to the gold‐standard reference (0.03 vs 0.07, −3.5 percentage point difference, 95% HDI: −8.0 to 0.8; 75.2% of the difference between protocols within ± 0.05 percentage points). For microbleeds, the fast scan was generally associated with a higher probability of assigning higher severity gradings than the standard acquisition.

Following joint image review and discussion, a case with ARIA‐E findings on a standard scan detected by one neuroradiologist was determined to be a false positive, in agreement with the gold‐standard assessment. In another case, distinguishing between white‐matter hyperintensities and ARIA‐E on the standard and fast scans required review of serial imaging; therefore, ARIA‐E could not be safely ruled out on the available scans alone. Results of the analysis using consensus diagnosis in place of the individual assessments were similar to those in the main analysis (see Table S3).

In terms of the interchangeability analysis, switching from standard to fast scans did not materially decrease agreement between neuroradiologists: ARIA‐E detection agreement was 0.95 within standard acquisitions versus 0.97 between protocols (−1.9 percentage point difference; 95% HDI –7.9 to 2.9; 83.9% of the difference between agreement estimates within ± 0.05 percentage points; see Table 2, Q3), and ARIA‐E severity agreement was 0.97 versus 0.95 (1.4 percentage point difference; 95% HDI −3.6 to 5.4; 96.0% of the difference between agreement estimates within ± 0.05 percentage points; see Q4).

**FIGURE 3:**
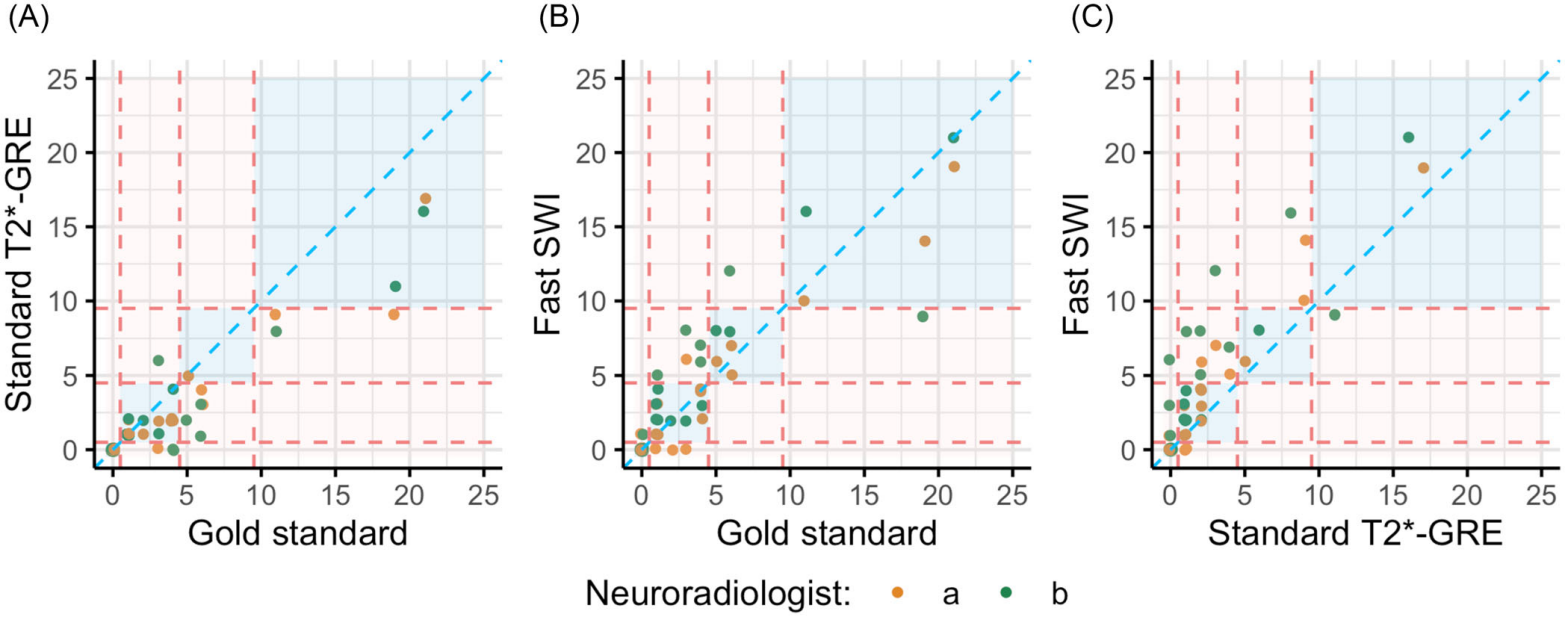
New microbleed counts from neuroradiologists’ assessments using standard and fast MRI sequences, and serial‐imaging gold‐standard assessments. Dashed coral lines indicate severity categories (mild, moderate, severe) and are based on the ARIA‐H microbleed radiographic severity scale. ARIA‐H, amyloid‐related imaging abnormalities with hemosiderin deposits; MRI, magnetic resonance imaging.

In terms of new microbleed counts, radiologists a and b identified new lesions in 16 and 15 standard scans, and in 15 and 18 fast scans. Observed counts ranged from 1–17 to 1–16 on standard scans, and 1–19 to 1–21 on fast scans. Figure 3 illustrates total microbleed counts on standard T2*‐GRE and fast SWI relative to the gold‐standard assessments, as well as the direct comparison of counts between fast SWI and standard T2*‐GRE (see Table S4 for further details). As expected, the incidence rate ratio for fast SWI relative to standard T2*‐GRE was 1.68 (95% HDI 1.31–2.07), indicating that fast SWI detected approximately 68% more microbleeds on average.

Finally, the two sequences were highly rated for image quality in both protocols (see Figure S1). Standard FLAIR quality scores among neuroradiologists were similar to those for fast FLAIR (standard: mean ± SD 4.4 ± 0.6; median [IQR] 4.5 [4.4–5.0]; fast: 4.2 ± 0.6; 4.2 [4.0–4.5]). Image quality for T2*‐GRE/SWI was also comparable between protocols (standard: 4.0 ± 0.7; 4.2 [3.5–4.5]; fast: 4.2 ± 0.8; 4.0 [4.0–5.0]). Incorporating image quality as a covariate did not meaningfully alter ARIA detection or severity metrics (see Table S5). All absolute differences in the expected log predictive density between nested models were smaller than 4, indicating similarly accurate predictions (see Table S6).^39^

## 4 DISCUSSION

In this study, we found that the fast 3D Wave‐CAIPI MRI protocol demonstrated performance comparable to contemporaneous standard 2D clinical trial acquisitions for the detection and grading of ARIA‐E, while substantially reducing scan time and improving spatial resolution. There were no false‐negative ARIA‐E cases and no meaningful differences in specificity of detection, which is highly encouraging for the potential use of accelerated 3D FLAIR in routine ARIA safety monitoring. Importantly, the absence of missed ARIA‐E cases supports the ability of such implementations to fulfil the safety‐critical role of radiological monitoring in both clinical trial and clinical practice settings. Although fast FLAIR showed a slight tendency to underestimate ARIA‐E severity in some cases, post hoc qualitative side‐by‐side review demonstrated concordant lesion localization between sequences. Where underestimation occurred, this was confined to cases in which small spatially separated hyperintensities contributed to higher ordinal severity grades on standard FLAIR. This likely reflects a combination of reduced conspicuity of subtle white matter hyperintensities on fast FLAIR and the use of standard acquisitions as the baseline comparator, which inherently favors that acquisition for interval change assessment.

The interchangeability analysis further suggests that standard 2D FLAIR can be replaced with fast 3D FLAIR without materially reducing agreement between neuroradiologists. The agreement metrics were high within and between protocols, with a 96% probability of the individual equivalence index for severity ratings lay within ± 0.05 percentage points. Consistent with these findings, image quality was rated as excellent or with only slight blurring in about 86% of scans (80%–92% across neuroradiologists), and image quality ratings did not meaningfully influence detection or severity estimates in our dataset. Nonetheless, the presence of subtle severity underestimation despite high image quality ratings suggests that optimization through post‐processing pipelines may further enhance performance. These results support further evaluation in larger studies.

Fast 3D FLAIR acquisitions offer several potential advantages over standard 2D FLAIR beyond time savings and multiplanar reconstruction. In multiple sclerosis, 3D FLAIR is more sensitive than 2D FLAIR for juxtacortical lesions,^40^ a location also relevant to ARIA‐E. This improvement is most likely related to reduced partial volume effects due to thinner slices in 3D acquisitions. Highly accelerated 3D FLAIR implementations (e.g., compressed sensing, Wave‐CAIPI) have also been shown to be non‐inferior to standard acquisitions in these regions.^28^, ^41^ Modern 3D FLAIR provides a more uniform cerebrospinal fluid suppression and substantially reduces pulsation/flow artifacts limiting false‐positive sulcal signal^42^, ^43^ that may mimic ARIA‐E. Isotropic datasets also enable advanced longitudinal registration and image subtraction algorithms,^44^ which can improve lesion conspicuity and differentiation from vascular or artifactual signal, and may increase sensitivity for ARIA‐E detection. Although our study did not implement such post‐processing methods, access to highly accelerated volumetric acquisitions lays the groundwork for future application. Improving early detection, radiological severity consistency, and inter‐rater agreement remain essential for successful DMT adoption at scale.

For microbleed detection, fast SWI and standard T2*‐GRE demonstrated similar sensitivity to detect cases with new microbleeds (both 0.91), although the wider posterior intervals compared to ARIA‐E reflect greater uncertainty in microbleeds estimates. As expected, the SWI detected more microbleeds that were missed with standard (T2*‐GRE) acquisitions, even when the assessments had the benefit of having serial MRI. The greater ability of SWI than T2*‐GRE to detect microbleeds is well documented.^11^, ^45^ Our results extend this evidence by showing that accelerated SWI retains this sensitivity advantage while providing excellent image quality and reducing acquisition time. Consistent with its higher sensitivity, fast SWI was less likely to underestimate microbleed severity than standard T2*‐GRE when compared to the gold‐standard serial assessments. This supports the role of SWI in improving detection of microbleeds. However, commonly used ARIA‐H severity thresholds originate from trials that used T2*‐GRE; therefore, the risk is less well established for SWI implementations. Furthermore, switching between acquisition types for ARIA‐H monitoring should be avoided and interpreted with caution.

Our study has several limitations. First, the sample size was limited and included only a small number of ARIA‐E cases; however, there were many scan pairs without ARIA‐E, reflecting a real‐world scenario. Second, given the limited cohort size, review of scans from the same participants may have introduced a degree of rater recall. To mitigate this, neuroradiologists were not informed of the image assessment design, including the number of sessions or scans per participant, ARIA prevalence, scan order, or time point, and scans from the same individual exhibited varying degrees of ARIA severity. Although sequence parameters were not displayed, differences between acquisitions are readily apparent on visual inspection. The inclusion of two standard scans introduces heterogeneity but reflects real‐world trade‐offs between spatial resolution, slice thickness, and acquisition time. Third, although serial imaging was used as a reference to provide a robust comparison, this benchmark was itself based on standard acquisitions, which could bias estimates in favor of that protocol. While this “gold‐standard” is not perfect, it represents the accepted reference in the field and provides a rigorous and conservative framework for comparison. In addition, it is possible that some microbleeds detected by fast SWI had been present at baseline and were not detected by the baseline T2*‐GRE sequence, reflecting known differences in sensitivity of SWI implementations; false‐positive findings cannot be entirely excluded. Fourth, the study was conducted on a single scanner and limited to one acceleration technique (Wave‐CAIPI); results may differ across platforms, coil configurations, sequence optimizations, and other acceleration methods. It should be noted that highly accelerated imaging techniques using artificial intelligence (AI) ‐based reconstruction methods are currently being developed by all the main MRI vendors (Philips' SmartSpeed, GE's AIR Recon DL and Siemens' Deep Resolve), opening up these fast‐scanning approaches more widely. Wave‐CAIPI SWI is available as a product sequence in Siemens software from ‘Syngo MR XA31’ onward. Fifth, we only assessed two admittedly key acquisitions for ARIA detection and safety monitoring. Diffusion imaging is also recommended in such protocols to differentiate from acute infarct; accelerated implementations of diffusion imaging, using parallel imaging and multi‐band acquisition schemes, are now available.^46^ Finally, no cases of superficial siderosis were detected in our cohort; therefore, the diagnostic performance of fast SWI for this ARIA‐H subtype could not be assessed. However, it would be expected to perform well based on its sensitivity to heme products.

In summary, our study suggests that fast MRI acquisitions enabled by Wave‐CAIPI can provide reliable ARIA‐E detection and severity gradings for the safety monitoring of DMTs in AD. These findings support further multi‐center research of accelerated MRI as a time‐efficient alternative for safety monitoring, particularly in settings where scan time, patient tolerance, or scanner availability are limiting factors in AD treatment. Research is needed to further evaluate the role of SWI (and highly accelerated implementations) for ARIA‐H safety monitoring.

## CONFLICT OF INTEREST STATEMENT

D. M. has no conflicts of interest. F. B. is a Data Safety Monitoring Board member for EISAI, Biogen, Prothena, and Merck. F. B. is a shareholder of Queen Square Analytics Limited. G. J. M. P. is a board member of and holds stock in Bioxydyn Limited, Queen Square Analytics Limited, and Quantitative Imaging Limited. H. R. C. has no conflicts of interest. H. M. is an employee of Siemens Healthineers. I. B. M. has a revenue‐sharing agreement for MIDAS software with UCLB. M.B. and M. R. G. have no conflicts of interest. N. C. F. has served on Data Safety Monitoring or Advisory Boards for Abbvie, Biogen, and F. Hoffmann‐La Roche. N. C. F. is a member of the Alzheimer's Society Research Strategy Council.

C. J. M. has received a grant award from Biogen for an investigator led trial of ultrafast MRI in dementia. C. J.M. has received consulting fees from Biogen, Roche, Lilly, Eisai, Novartis, Neuoimmune, MSD and GSK. C. J. M. has received honoraria for lecturing at scientific symposia sponsored by Eisai and Lilly. C. J.M. has received support for attending meetings or travel from Biogen, Roche, Eisai and Alector. C. J. M. has serve on the Data Safety Monotoring Board for Imperial and Immunobrain and is the Director for NIHR UK Dementia Trials Network. C. T. is an employee of Siemens Healthcare. D. L. T. has received support from the Alzheimer's Society. F. B. has grants or contracts with EPSRC, EU‐JU (IMI), NIHR‐BRC, GEHC and ADDI, and has received consulting fees from Combinostics, IXICO, Roche, Scottish Brain Sciences. G. J. M. P. has received grant funding from Alzheimer's Society, Biogen, Siemens Healthineers and Eli Lilly. I. B.M. has received grants from NIH, Wolfson Foundation, and ARUK. N. C. F. has received consulting fees or honoraria from Eisai, F. Hoffmann‐La Roche, and Eli Lilly. N.M. is supported by NIHR UCLH BRC.W. L. is an employee of Siemens Healthineers.

## CONSENT STATEMENT

All human subjects involved in this study provided written informed consent.

## Supporting information



Supporting Information

Supporting Information
