# Supplementary material for: Accelerated 3D MRI for ARIA monitoring in Alzheimer's disease
Source: Alzheimers Dement. 2026 Mar 24;22(3):e71297. doi: 10.1002/alz.71297 (PMC13093291; doi:10.1002/alz.71297)
Supplement: Supplementary file 1 — Supporting Information [file ALZ-22-e71297-s002.docx]

Supplementary Table 1: Image evaluation questionnaire.

| ARIA Radiographic Severity Score | | | |
| --- | --- | --- | --- |
|  | Mild | Moderate | Severe |
| ARIA-E: new emergent sulcal and/or cortical/subcortical FLAIR hyperintensity and gyral swelling | One location < 5 cm | One location 5-10 cm or more than one location each < 10 cm | One or more location > 10 cm |
| ARIA-H: new emergent microbleeds | ≤ 4 | 5-9 | ≥ 10 |
| ARIA-H: new emergent superficial siderosis | 1 focal area | 2 focal areas | > 2 focal areas |
| Image Quality Score | | | |
| 1 | Non-diagnostic featuring strong artefacts | | |
| 2 | Severe blurring rendering evaluation uncertain | | |
| 3 | Moderate blurring that slightly compromises assessment | | |
| 4 | Slight blurring that did not compromise image assessment | | |
| 5 | Excellent without artefacts | | |

Supplementary Table 2: Statistical analysis details.

| We ran four chains of 4,000 iterations each (1,000 warm-up). Student-t priors with 3 degrees of freedom, mean 0, and scale 2.5 were used for intercepts and random effects, and normal priors with mean 0 and scale 2.5 were used for fixed effect coefficients. Priors were examined to confirm they were weakly informative relative to the scale of the data. Model comparisons were performed with the 'loo' (version 2.8.0.9000) package. Summary statistics are based on predictions conditional on the estimated group-specific random effects for the observed groups. Convergence was assessed with R̂ (< 1.01) and effective sample sizes (> 1,000). For the microbleed count analysis, overdispersion was assessed using simulation-based residual diagnostics ('DHARMa', version 0.4.7), including the dispersion test, and a negative binomial model was considered but not needed. Plots were generated using the 'ggplot2' (version 3.5.2) and the 'likert' (version 1.3.5.1) packages. |
| --- |

Supplementary Table 3: ARIA detection and severity for standard and fast protocols based on consensus diagnosis.

| **Q1. How accurately did standard and fast scans detect presence/absence of ARIA relative to to serial-imaging gold-standard assessments?** | | | | | |
| --- | --- | --- | --- | --- | --- |
| Type of ARIA |  | Standard scans* | Fast scans* | % Difference  (95% HDI) | % in ROPE |
| ARIA-E | Sensitivity | 1.00 | 1.00 | 0.0 (0.0, 0.0) | 100.0 |
|  | Specificity | 0.94 | 0.94 | -0.0 (-5.3, 5.1) | 94.2 |
| ARIA-H (microbleeds) | Sensitivity | 0.95 | 0.99 | 2.5 (-7.4, 15.2) | 65.9 |
|  | Specificity | 0.99 | 0.92 | -5.6 (-16.4, 2.6) | 43.9 |
| **Q2. How consistent were ARIA severity ratings from standard and fast scans relative to serial-imaging gold-standard assessments?** | | | | | |
| Type of ARIA |  | Standard scans* | Fast scans* | % Difference  (95% HDI) | % in ROPE |
| ARIA-E | Lower severity | 0.04 | 0.07 | 2.7 (-3.5, 9.9) | 75.1 |
|  | Same severity | 0.90 | 0.89 | -0.9 (-6.6, 2.6) | 92.4 |
|  | Higher severity | 0.06 | 0.05 | -1.2 (-6.3, 2.0) | 94.2 |
| ARIA-H (microbleeds) | Lower severity | 0.09 | 0.02 | -6.7 (-14.3, -0.6) | 30.2 |
|  | Same severity | 0.89 | 0.87 | -1.3 (-10.0, 7.1) | 76.3 |
|  | Higher severity | 0.02 | 0.11 | 8.3 (1.4, 16.8) | 17.7 |
| NOTE. *Values represent posterior medians on the probability scale from Bayesian logistic mixed-effects models. In terms of detection against the gold standard (Q1), there were no false-negatives for ARIA-E for standard and fast scans. | | | | | |
| Abbreviations: ARIA, amyloid-related imaging abnormalities; ARIA-E, amyloid-related imaging abnormalities with edema/effusion; ARIA-H, amyloid-related imaging abnormalities with hemosiderin deposits; HDI, high density interval. ROPE, region of practical equivalence. | | | | | |

Supplementary Table 4: Number of scans stratified by ARIA-H severity.

| Rater | Acquisition | ARIA-H severity | N (proportion)* |
| --- | --- | --- | --- |
| Rater a | Standard T2*-GRE | 0 (No new lesions) | 24 (0.60) |
| Rater b |  |  | 25 (0.62) |
| Rater a | Fast SWI |  | 25 (0.62) |
| Rater b |  |  | 22 (0.55) |
| Rater a | Standard T2*-GRE | 1 (Mild) | 12 (0.30) |
| Rater b |  |  | 11 (0.28) |
| Rater a | Fast SWI |  | 8 (0.20) |
| Rater b |  |  | 8 (0.20) |
| Rater a | Standard T2*-GRE | 2 (Moderate) | 3 (0.07) |
| Rater b |  |  | 2 (0.05) |
| Rater a | Fast SWI |  | 4 (0.10) |
| Rater b |  |  | 7 (0.17) |
| Rater a | Standard T2*-GRE | 3 (Severe) | 1 (0.03) |
| Rater b |  |  | 2 (0.05) |
| Rater a | Fast SWI |  | 3 (0.07) |
| Rater b |  |  | 3 (0.07) |
| NOTE. *Number of scans (and their proportion), stratified by rater, acquisition type, and ARIA-H (microbleeds) severity. | | | |
| Abbreviations: ARIA-H, amyloid-related imaging abnormalities with hemosiderin deposits. | | | |


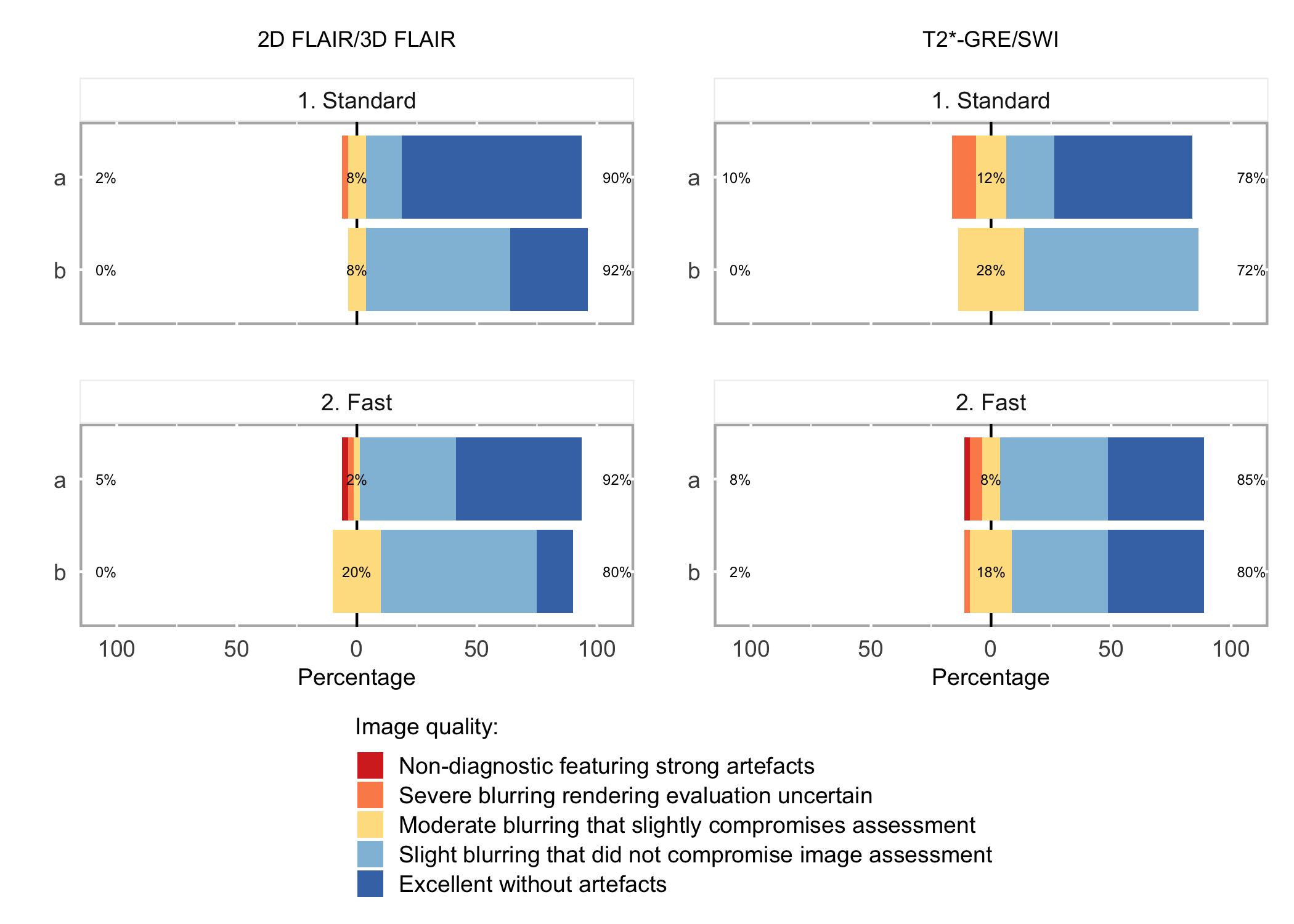


Supplementary Figure 1: Image quality scores per protocol and neuroradiologist (a and b). Percentages of scans are displayed as a diverging stacked barplot, with non-diagnostic and severe blurring categories on the left, moderate blurring in the middle, and slight blurring and excellent quality on the right.

Supplementary Table 5: ARIA detection and severity for standard and fast protocols incorporating image quality as a covariate.

| **Q1. How accurately did standard and fast scans detect presence/absence of ARIA relative to serial-imaging gold-standard assessments?** | | | | | |
| --- | --- | --- | --- | --- | --- |
| Type of ARIA |  | Standard scans* | Fast scans* | % Difference  (95% HDI) | % in ROPE |
| ARIA-E | Sensitivity | 1.00 | 1.00 | 0.0 (0.0, 0.0) | 100.0 |
|  | Specificity | 0.94 | 0.95 | -0.8 (-5.1, 2.8) | 97.3 |
| ARIA-H (microbleeds) | Sensitivity | 0.91 | 0.92 | -0.7 (-12.0, 9.9) | 64.7 |
|  | Specificity | 0.99 | 0.96 | 2.8 (-1.0, 8.8) | 79.1 |
| **Q2. How consistent were ARIA severity ratings from standard and fast scans relative to serial-imaging gold-standard assessments?** | | | | | |
| Type of ARIA |  | Standard scans* | Fast scans* | % Difference  (95% HDI) | % in ROPE |
| ARIA-E | Lower severity | 0.03 | 0.07 | -3.4 (-7.8, 0.8) | 76.6 |
|  | Same severity | 0.91 | 0.90 | 0.8 (-1.9, 4.3) | 98.4 |
|  | Higher severity | 0.06 | 0.03 | 2.4 (-0.3, 5.5) | 94.3 |
| ARIA-H (microbleeds) | Lower severity | 0.14 | 0.06 | 8.4 (1.9, 15.3) | 14.5 |
|  | Same severity | 0.83 | 0.85 | -1.3 (-7.5, 4.9) | 86.4 |
|  | Higher severity | 0.02 | 0.09 | -7.0 (-12.1, -2.4) | 20.0 |
| **Q3. Can standard and fast scans be used interchangeably for the detection of ARIA?** | | | | | |
| Type of ARIA |  | Within Standard Acquisitions* | Between Protocols* | % Difference†  (95% HDI) | % in ROPE |
| ARIA-E | Agreement | 0.95 | 0.97 | -1.8 (-7.5, 2.8) | 85.4 |
| **Q4. Can standard and fast scans be used interchangeably for grading ARIA severity?** | | | | | |
| Type of ARIA |  | Within Standard Acquisitions* | Between Protocols* | % Difference†  (95% HDI) | % in ROPE |
| ARIA-E | Agreement | 0.97 | 0.95 | 1.4 (-3.5, 5.4) | 95.9 |
| NOTE. *Values represent posterior medians on the probability scale from Bayesian logistic mixed-effects models incorporating image quality as a covariate. †Individual equivalence index. In terms of detection against the gold standard (Q1), there were no false-negatives for ARIA-E for standard and fast scans. | | | | | |
| Abbreviations: ARIA, amyloid-related imaging abnormalities; ARIA-E, amyloid-related imaging abnormalities with edema/effusion; ARIA-H, amyloid-related imaging abnormalities with hemosiderin deposits; HDI, high density interval; ROPE, region of practical equivalence. | | | | | |

Supplementary Table 6: Expected Log Predictive Density Comparisons of Nested Models.

| **Q1.** | | |
| --- | --- | --- |
| Type of ARIA | Models* | \| ELPD Difference \| |
| ARIA-E | Scan type * Rater + Image quality score | 1.0 |
| ARIA-H (microbleeds) | Scan type * Rater + Image quality score | 0.3 |
| **Q2.** | | |
| Type of ARIA | Models* | \| ELPD Difference \| |
| ARIA-E | Scan type * Rater + Image quality score | 0.1 |
| ARIA-H (microbleeds) | Scan type * Rater + Image quality score | 2.4 |
| **Q3.** | | |
| Type of ARIA | Models* | \| ELPD Difference \| |
| ARIA-E | Scan pair * Rater pair + Difference in image quality scores | 0.1 |
| **Q4.** | | |
| Type of ARIA | Models* | \| ELPD Difference \| |
| ARIA-E | Scan pair * Rater pair + Difference in image quality scores | 0.6 |
| NOTE. *Comparison with simple model without image quality as a covariate. | | |
| Abbreviations: ARIA, amyloid-related imaging abnormalities; ARIA-E, amyloid-related imaging abnormalities with edema/effusion; ARIA-H, amyloid-related imaging abnormalities with hemosiderin deposits; ELPD, Expected Log Predictive Density | | |
